# Osimertinib early dose reduction as a risk to brain metastasis control in EGFR‐mutant non‐small cell lung cancer

**DOI:** 10.1002/cam4.6393

**Published:** 2023-09-11

**Authors:** Takehiro Tozuka, Rintaro Noro, Akihiko Miyanaga, Shinji Nakamichi, Susumu Takeuchi, Masaru Matsumoto, Kaoru Kubota, Kazuo Kasahara, Masahiro Seike

**Affiliations:** ^1^ Department of Pulmonary Medicine and Oncology, Graduate School of Medicine Nippon Medical School Tokyo Japan

**Keywords:** brain metastases, dose reduction, non‐small cell lung cancer, osimertinib

## Abstract

**Background:**

The epidermal growth factor receptor (EGFR) mutation is a risk factor associated with brain metastases (BMs) in patients with non‐small cell lung cancer (NSCLC). This study aimed to evaluate the impact of osimertinib early dose reduction on BM worsening.

**Methods:**

We retrospectively analyzed EGFR‐mutant NSCLC patients treated with osimertinib as first‐line treatment between August 2018 and October 2021. To evaluate the impact of osimertinib early dose reduction, we performed a landmark analysis of patients who achieved disease control at 4 months. Patients were divided into two groups according to whether the osimertinib dose was reduced or not, within 4 months after the start of treatment. We evaluated the time to BMs onset or progression, progression‐free survival, and overall survival.

**Results:**

In total, 62 NSCLC patients with EGFR mutations were analyzed. Thirteen patients experienced early dose reduction of osimertinib treatment. Seven patients received osimertinib 40 mg daily, and six received 80 mg every other day. The most common reason for dose reduction was gastrointestinal toxicity (*n* = 4), followed by skin rashes (*n* = 3). The time to BMs onset or progression was significantly shorter in patients who experienced early dose reduction than in those who continued regular treatment (Hazard ratio 4.47, 95% confidence interval, 1.52–13.11). The 1‐year cumulative incidence of BM onset or progression was 23.1% in the reduced‐dose group and 5.0% in the standard dose group. The risk of worsening BMs with early dose reduction of osimertinib treatment was higher in patients who had BMs before treatment and in younger patients.

**Conclusion:**

Early dose reduction of osimertinib was a risk factor for the worsening of BMs. A higher risk was associated with younger patients and those presenting BMs before treatment.

## 1 INTRODUCTION

Lung cancer is the leading cause of cancer‐related death worldwide, with non‐small cell lung cancer (NSCLC) accounting for approximately 80% of all lung cancers.^1^, ^2^ Lung cancer often develops brain metastases (BMs) at the time of initial diagnosis, and 20%–40% of patients with NSCLC develop BMs during the treatment course.^3^, ^4^ Furthermore, patients with NSCLC and positive epidermal growth factor receptor (EGFR) mutations have a higher incidence of BM than those with wild‐type EGFR.^5^

EGFR tyrosine kinase inhibitors (TKIs) have significantly improved the prognosis of patients with EGFR‐mutant NSCLC. In the FLAURA trial, osimertinib prolonged progression‐free survival (PFS) and overall survival (OS) compared with first‐generation EGFR TKIs such as gefitinib or erlotinib in patients with previously untreated EGFR‐positive NSCLC.^6^ Therefore, osimertinib has become the first‐line standard treatment for EGFR‐mutant advanced NSCLC. Osimertinib is also more effective than other EGFR TKIs against BMs. A preclinical study evaluating the brain penetration and activity of osimertinib in animal models showed that under positron emission tomography micro‐dosing conditions, the brain had greater exposure to osimertinib than other EGFR TKIs.^7^ In the FLAURA trial, central nervous system (CNS) PFS was significantly longer in the osimertinib group than in the first‐generation EGFR TKIs group.^8^ In the ADAURA trial, a phase 3 trial comparing osimertinib with a placebo in patients with completely resected EGFR‐mutant NSCLC, the CNS disease‐free survival was significantly longer in the Osimertinib group than that of the placebo.^9^

Despite these results, some patients treated with osimertinib still experience BM progression during treatment. Management of BMs is crucial due to their unfavorable impact on patients' quality of life (QOL) compared to other metastatic sites.^10^ Moreover, BMs can negatively influence the following treatment strategy because the presence of the blood–brain barrier (BBB) limits the efficacy of anticancer agents in the CNS. The BBB restricts drug penetration into the CNS and promotes drug removal by expressing multidrug‐resistant transporters and P‐glycoprotein.^11^ Therefore, it is essential to identify the factors associated with BM worsening during treatment in patients with EGFR‐positive lung cancer.

In the FLAURA trial, osimertinib showed a similar safety profile and lower rates of serious adverse events (AEs) than the first‐generation EGFR TKIs.^6^ Although osimertinib's AEs are relatively manageable, 4% of treated patients in the FLAURA trial experienced AEs leading to dose reduction, and 13% needed to be discontinued. In the clinical practice, the osimertinib dose may be reduced due to AEs such as skin rash, gastrointestinal toxicity, and cardiac toxicities. Dose reductions may decrease blood concentrations, resulting in less translocation to the cranial spinal fluid.^7^, ^12^ The present study aimed to identify the risk of BM worsening in patients with EGFR‐positive NSCLC treated with osimertinib, specifically to evaluate the impact of osimertinib dose reduction on BM control.

## 2 METHODS

### 2.1 Patients and data collection

We retrospectively reviewed patients' medical records at Nippon Medical School Hospital (Tokyo, Japan) between August 2018 and October 2021. The present study included patients with pathologically confirmed advanced NSCLC with EGFR‐positive mutation who received osimertinib as first‐line treatment. In the present study, patients presenting EGFR exon 19 deletion (Ex19del) and EGFR exon 21 Leu858Arg (Ex 21 L858R) mutations, as well as other uncommon EGFR mutations, were included. The study protocol was approved by the Ethics Committee of Nippon Medical School (approval number B‐2022‐544). Because of the retrospective nature of the study, the need for informed patient consent was waived.

### 2.2 Assessment and analysis

All patients were examined for BMs using head computer tomography (CT) or magnetic resonance imaging (MRI) prior to the start of osimertinib treatment. Radiological evaluations were performed at the discretion of the attending physician in the clinical practice; if performed, they were continued approximately every 6–10 weeks. The patients were generally seen approximately every month and evaluated by the attending physician for BM symptoms. A landmark analysis was performed to assess the impact of osimertinib dose reduction. The final analysis included patients who were able to continue osimertinib treatment and showed disease control for at least 4 months. We divided the patients into two groups based on the need for dose reduction. The early dose reduction group included patients who underwent a dose reduction within 4 months after the start of osimertinib treatment. The standard dose group included patients who continued regular treatment with no dose reduction within the same period. We compared the time to BM worsening, PFS, and OS between the two groups. Evaluation of BM worsening was performed according to the Response Assessment in Neuro‐Oncology Brain Metastases (RANO‐BM) criteria.^13^

### 2.3 Statistical analysis

Patient characteristics between the two groups were compared using the Mann–Whitney U test or Fisher's exact test. The probability of BM worsening was estimated using cumulative incidence functions, and Gray's test was performed to compare the associated risk between the different groups. Fine‐Gray models were used to determine hazard ratios (HRs) for time to BM worsening. All factors presenting *p* < 0.10 in the univariate analysis were tested in a multivariate analysis. Kaplan–Meier curves for PFS and OS were generated. We performed a log‐rank test to assess the differences in PFS and OS between the different groups. Cox proportional hazard models were used to determine the HR for PFS and OS. Any *p* < 0.05 was considered statistically significant. All statistical analyses were performed using EZR® version 1.55 software (Saitama Medical Center, Jichi Medical University, Saitama, Japan).^14^

## 3 RESULTS

**FIGURE 1:**
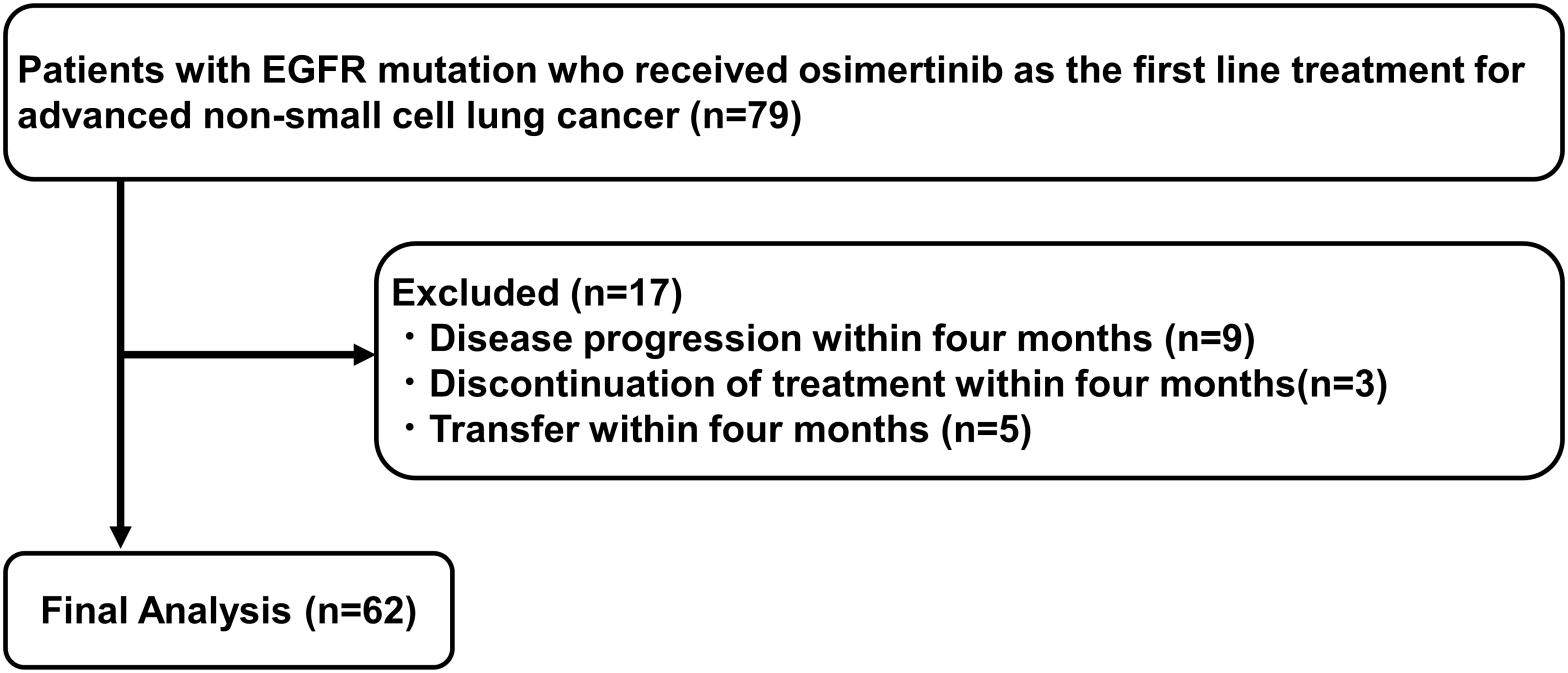
Patient selection flow.

The patient selection flowchart is shown in Figure 1. A total of 62 NSCLC patients with EGFR mutations were analyzed. The median follow‐up time was 24.0 months (95% confidence interval [CI], 16.9–27.0 months) (Kaplan–Meier estimate).

Table 1 displays the patient characteristics. The median age was 73 years (range 34–89). Most patients had a performance status (PS) of 0 or 1. All patients were diagnosed with adenocarcinoma, and almost 30% showed evidence of BM before the osimertinib treatment. No patients with leptomeningeal metastases were included in the present study. Among the 62 patients, 35 presented EGFR Ex19del, 22 Ex21 L858R, and six EGFR uncommon mutations. The standard dose group included 49 patients, while the early dose reduction group included 13 patients. Seven of these 13 patients received 40 mg osimertinib daily, and six received 80 mg osimertinib every other day. The most common reason for osimertinib dose reduction was gastrointestinal toxicity, such as grade (Gr) 2 anorexia (*n* = 3) and Gr2 diarrhea (*n* = 1), followed by Gr2 skin rash (*n* = 3). Other reasons for dose reduction were Gr4 Creatine phosphokinase elevation (*n* = 1), Gr2 QT prolongation (*n* = 1), Gr2 thrombocytopenia (*n* = 1), Gr2 liver dysfunction (*n* = 1), and at the patient's request (*n* = 2). There were no significant differences in the background factors between the standard dose and early dose reduction groups.

**TABLE 1:** Patient characteristics.

	All patients (*n* = 62)	Standard dose group (*n* = 49)	Early dose reduction group (*n* = 13)	*p*‐value
Age, median (range)	73 (34–89)	72 (50–85)	77 (34–89)	0.446
<75 years	34 (54.8%)	29 (59.2%)	5 (38.5%)	0.220
≥75 years	28 (45.2%)	20 (40.8%)	8 (61.5%)	
Sex				
Male	23 (37.1%)	18 (36.7%)	5 (38.5%)	1.000
Female	39 (62.9%)	31 (63.3%)	8 (61.5%)	
Performance status				
0, 1	55 (88.7%)	43 (87.8%)	12 (92.3%)	1.000
2–4	7 (11.3%)	6 (12.2%)	1 (7.7%)	
Smoking status				
Current/Former	24 (38.7%)	18 (36.7%)	6 (46.2%)	0.541
Never	38 (61.3%)	31 (63.3%)	7 (53.8%)	
Histology				
Adenocarcinoma	62 (100%)	49 (100%)	13 (100%)	NA
Non‐adenocarcinoma	0 (0%)	0 (0%)	0 (0%)	
EGFR				
Exon 19 del	35 (56.5%)	24 (49.0%)	11 (84.6%)	0.086
Exon 21 L858R	22 (35.5%)	20 (40.8%)	2 (15.4%)	
Uncommon	5 (8.0%)	5 (10.2%)	0 (0%)	
Body surface area (m^2^), median (range)	1.45 (1.06–1.75)	1.60 (1.06–1.99)	1.49 (1.19–1.75)	0.139
Brain metastases before treatment				
Yes	17 (27.4%)	13 (26.5%)	4 (30.8%)	0.739
No	45 (72.6%)	36 (73.5%)	9 (69.2%)	

Abbreviations: EGFR, epidermal growth factor receptor; Ex 19del, exon 19 deletion; Ex 21L858R, exon 21 L858R point mutation; NA, not available.

**FIGURE 2:**
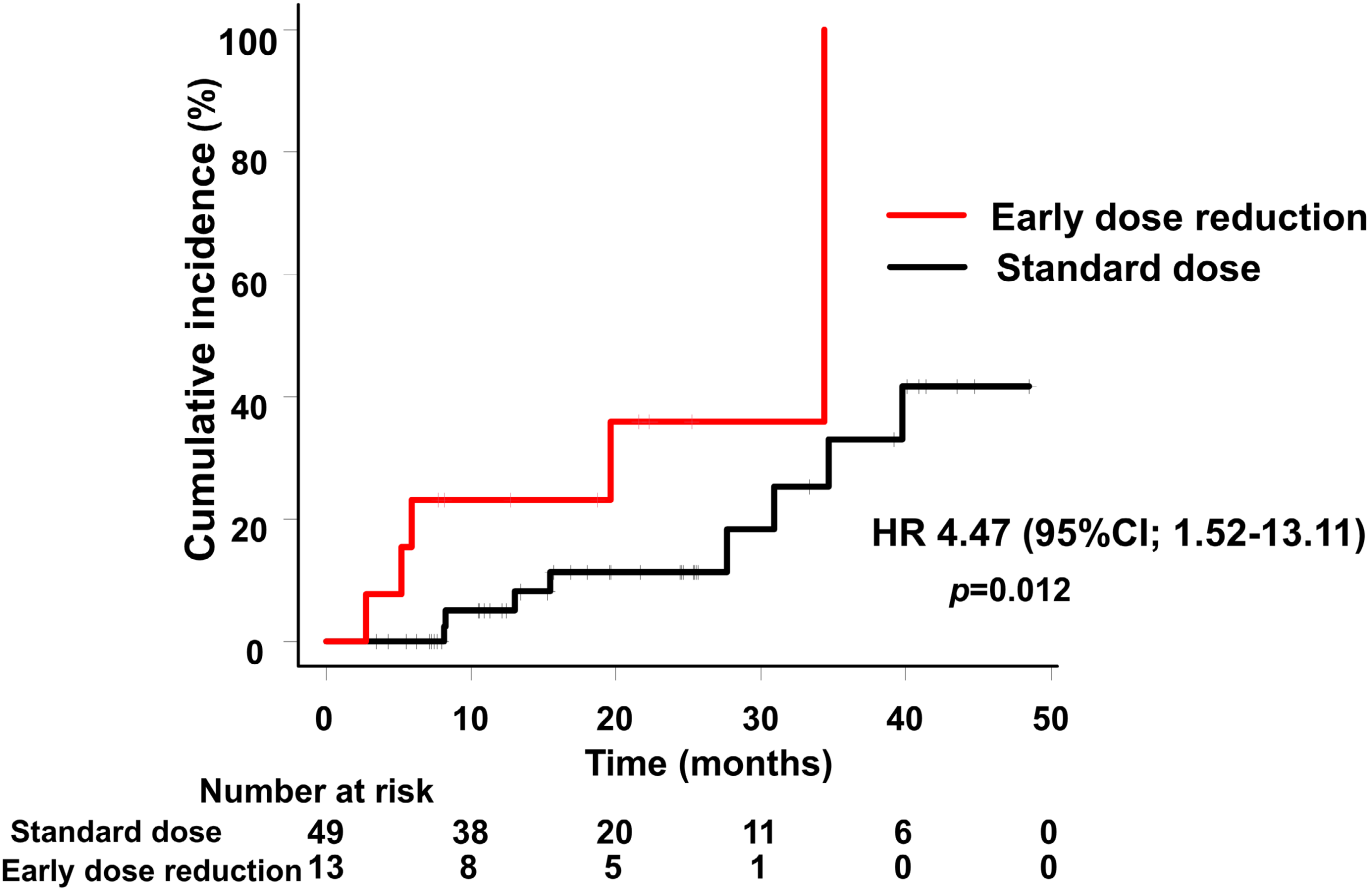
Time to brain metastasis onset or progression (Early dose reduction group vs. standard dose group).

The time to BM onset or progression was significantly shorter in the early dose reduction group than in the standard dose group (HR 4.47, 95% CI, 1.52–13.11) (Figure 2). The 1‐year cumulative incidence of the BM onset or progression was 23.1% in the early dose reduction group and 5.0% in the standard dose group, while the 2‐year‐cumulative incidence was 35.9% and 11.4%, respectively.

Several factors were identified as related to BM onset or progression (*p* < 0.10) by univariate analysis: age <75 years old, female sex, BM occurrence before osimertinib treatment, and osimertinib early dose reduction. When evaluating these factors by multivariate analysis, results showed that osimertinib early dose reduction was a significantly associated risk factor, independent of the presence of BMs before osimertinib treatment (Table 2). There was no significant difference in the time to BM worsening between patients with EGFR 19del and those with EGFR L858R mutations (Figure S1).

**TABLE 2:** Univariate and multivariate analyses of factors associated with brain metastasis onset and progression.

Variables	Univariate analysis			Multivariate analysis		
	HR	95% CI	*p*‐value	HR	95% CI	*p*‐value
Age (years)						
<75 versus ≥75	4.53	0.99, 20.77	0.052	4.65	0.59, 36.78	0.150
Sex						
Male versus female	0.27	0.06, 1.17	0.080	1.39	0.21, 8.59	0.760
Performance status						
0–1 versus 2–4	2.40	0.29, 19.76	0.420			
Smoking						
Current/Ex versus never	1.74	0.59, 5.11	0.310			
Body surface area (m^2^)						
≥1.5 versus <1.5	1.35	0.45, 4.02	0.590			
EGFR status						
Common versus uncommon	1.61	0.18, 14.68	0.670			
Ex19del versus Ex21 L858R	1.27	0.39, 4.15	0.690			
Brain metastases before treatment						
Yes versus no	8.72	2.94, 25.90	<0.001	12.57	1.59, 99.40	0.016
Group						
Early dose reduction versus standard dose	4.47	1.52, 13.11	0.006	12.79	1.98, 82.47	0.007

Abbreviations: CI, confidence interval; EGFR, epidermal growth factor receptor; Ex 19del, exon 19 deletion; Ex 21L858R, exon 21 L858R point mutation; HR, hazard ratio.

**FIGURE 3:**
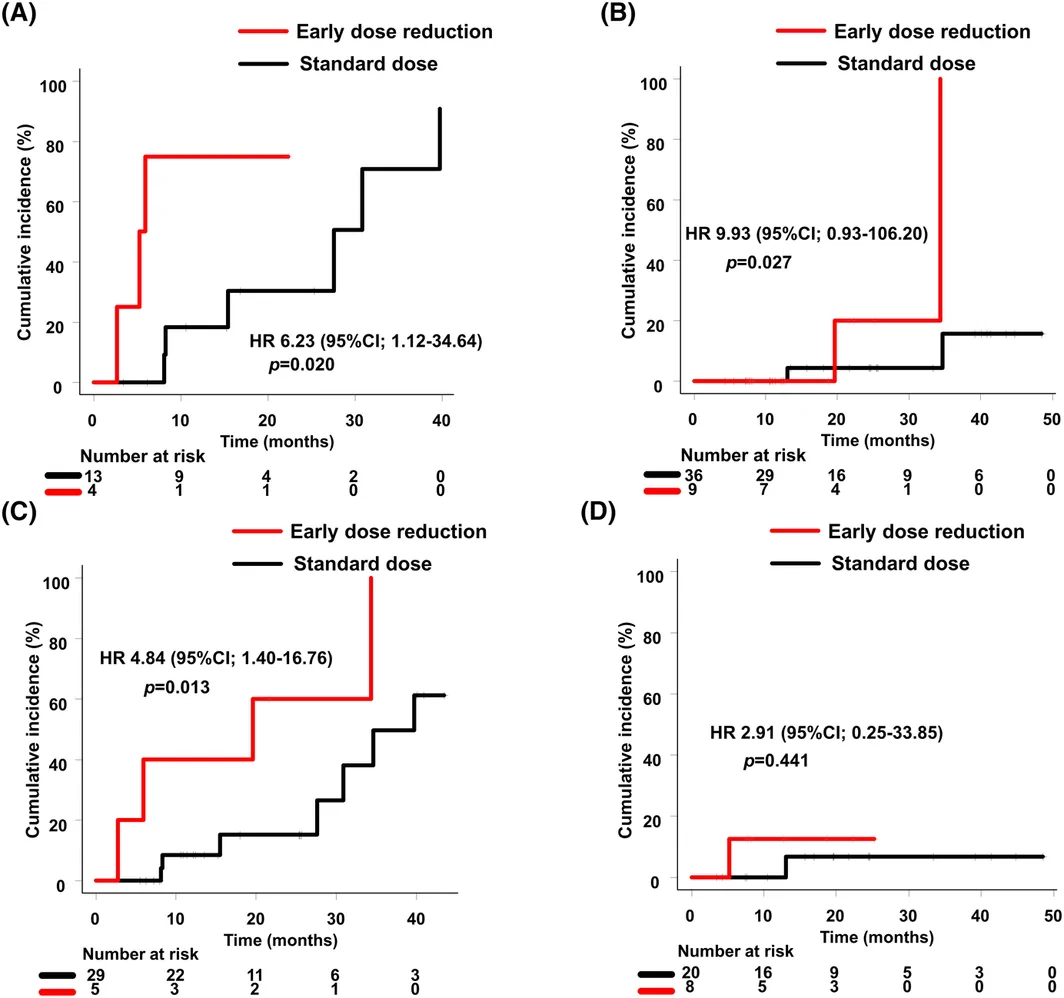
Time to brain metastasis onset or progression (Early dose reduction group vs. standard dose group): (A) patients with brain metastases before treatment; (B) patients without brain metastases before treatment; (C) patients aged <75 years old; (D) patients aged ≥75 years old.

The subgroup analysis of patients presenting BM prior to the start of treatment suggested that patients in the early dose reduction group showed higher cumulative worsening incidence, meaning that BMs would progress more rapidly. Cumulatively, over 1 year, the BM incidence was 75% in the early dose reduction group and 18.2% in the standard dose group (Figure 3A). On the other hand, the subgroup analysis of patients without BMs prior to osimertinib treatment showed a cumulative incidence over 2 years of 20% in the early dose reduction group (Figure 3B). When considering patients under 75 years of age, the time to the BM onset or progression was significantly shorter in the early dose reduction group compared with the standard dose group (HR 4.84, 95% CI, 1.40–16.76) (Figure 3C), while there was no significant difference between the two groups in patients ≥75 years old (HR 2.91, 95% CI 0.25–33.85) (Figure 3D).

**FIGURE 4:**
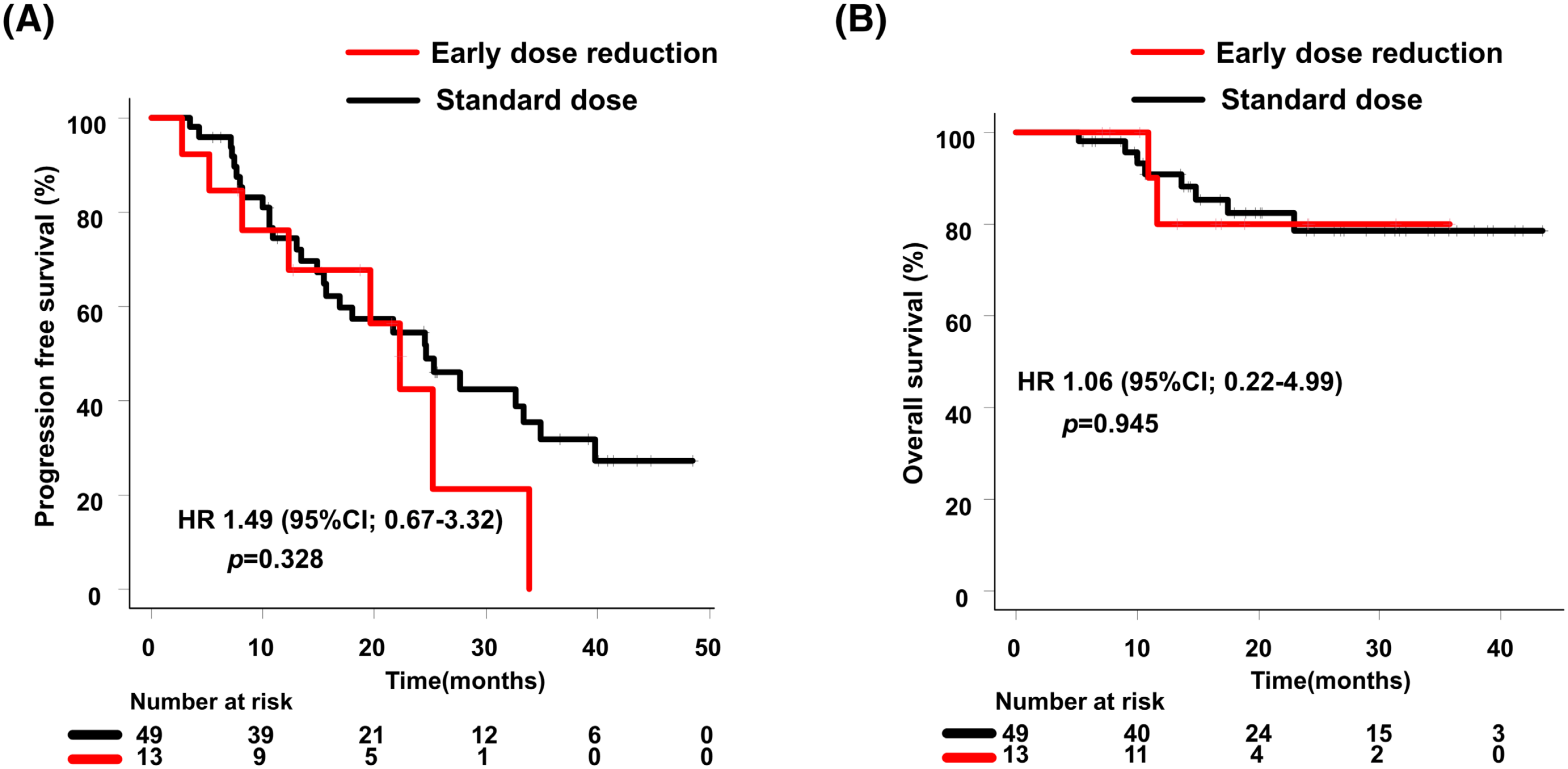
(A) Progression‐free survival (PFS); (B) Overall survival (OS) (Early dose reduction group vs. standard dose group).

PFS of patients in the early dose reduction group was not significantly different from that of patients in the standard dose group (median, 22.3 months vs. 24.6 months; HR 1.49 [95% CI, 0.67–3.32]) (Figure 4A). OS was also not significantly different between the two groups (HR 1.06 [95% CI, 0.22–4.99]) (Figure 4B).

## 4 DISCUSSION

The present study showed that osimertinib early dose reduction has a negative effect on the control of BMs. In particular, patients with BMs before osimertinib treatment or patients <75 years old had a higher risk of BM onset and progression after the reduction of osimertinib dose.

The results of this study support the possibility that osimertinib's cerebrospinal fluid (CSF) levels in patients receiving a reduced dose may not be sufficient to suppress cancer cells in the CNS. CSF concentration of EGFR TKIs depends on their concentration in blood. A previous study reported that the concentration of gefitinib in the spinal fluid increased from 18 to 42 nmol/L when the dose was increased from 500 mg to 1000 mg in patients with leptomeningeal metastases, resulting in the disappearance of cancer cells in the spinal fluid.^15^ Similarly, weekly intermittent “pulses” of high‐dose erlotinib (1500 mg) resulted in CSF concentrations above half‐maximal inhibitory concentrations against lung cancer cells with EGFR mutations in patients with leptomeningeal metastases.^16^ Pulsatile erlotinib for CNS progressed metastases achieved 67% (6/9) radiological response rates. In patients with EGFR‐mutant NSCLC showing CNS progression on 80 mg/day osimertinib, dose escalation to 160 mg resulted in moderate efficacy, with CNS control lasting approximately between 3 and 6 months.^17^ These studies showed that TKI dose might be important for BM control, even in patients treated with osimertinib. A patient's body surface area can also affect drug concentration in the blood, but this was not identified as a significant risk factor for BM worsening in the present study. Considering that gastrointestinal and skin toxicity led to osimertinib dose reduction in this work, managing side effects appropriately becomes essential for BM control.

In line with our results and despite differences in age definitions, several studies have revealed that younger age is a risk factor for BMs in patients with EGFR‐mutant NSCLC.^18^, ^19^, ^20^, ^21^ Biological factors may differ between younger and older patients, including high levels of Ki‐67 and elevated expression of vascular endothelial growth factor (VEGF).^4^, ^22^ Extensive BBB dysfunction associated with aging may also be a reason for the differences associated with BM risk during treatment.^23^ Since the BBB is more robust in younger patients, the same drug dose may not be sufficient to be transferred into the CSF as in older patients.

EGFR mutation type did not affect the BM risk in the present study. EGFR Ex 19del or Ex 21 L858R may be different prognostic markers for patients treated with EGFR TKIs. The LUX‐Lung 3 and LUX‐Lung 6 trials suggested that afatinib prolonged OS compared with chemotherapy in patients with EGFR Ex 19del but not in patients with EGFR Ex 21 L858R.^24^ A previous study examined BM risk factors based on the EGFR mutation type in patients with NSCLC treated with gefitinib or erlotinib and concluded that EGFR Ex 21 L858R was an independent risk factor.^18^ Another study concluded that patients with NSCLC who received gefitinib or erlotinib and presented EGFR Ex 19del mutation were at a higher risk of CNS progression.^25^ As evidenced, the impact of EGFR mutation subtypes on BM worsening is still controversial. Regarding osimertinib treatment, the FLAURA study showed that it consistently prolonged PFS over first‐generation TKIs in patients with EGFR Ex 19del and EGFR Ex 21 L858R.^6^ Therefore, EGFR mutations may not be an associated risk factor for the worsening of BMs in patients treated with osimertinib.

When considering effectiveness variables, PFS was not significantly different between groups, and OS results were immature at the time of analysis. Several clinical trials have investigated the efficacy of EGFR TKI dose reductions. Afatinib administered at a starting daily dose of 20 mg, showed a PFS of 12.6–14.2 months,^26^, ^27^ similar to that reported in other clinical trials at a standard daily dose of 40 mg.^28^, ^29^ Indeed, TKI dosage may have little effect on the progression of extracranial diseases. Blood TKI concentrations required to control extracranial lesions may differ from those needed to control intracranial lesions since the BBB acts as a barrier to drug delivery to the brain. Further studies are required to determine the clinical efficacy of low‐dose osimertinib.

The limitations of this study are its single‐center retrospective design and the small sample size. The results would need to be validated by future large‐scale studies. Few studies have evaluated the effect of EGFR TKI dose reduction on the control of BMs. The present study provides valuable insight into BM management in patients with EGFR‐positive NSCLC.

In conclusion, early dose reduction of osimertinib was a risk factor for BM worsening, independent of the presence of BMs before treatment. A higher risk was associated with younger patients and those presenting BMs before treatment.

## AUTHOR CONTRIBUTIONS

**Takehiro Tozuka:** Conceptualization (lead); data curation (lead); formal analysis (lead); methodology (lead); visualization (lead); writing – original draft (lead). **Rintaro Noro:** Conceptualization (supporting); formal analysis (equal); methodology (supporting); resources (equal); writing – review and editing (lead). **Akihiko Miyanaga:** Data curation (supporting); investigation (supporting); writing – review and editing (supporting). **Shinji Nakamichi:** Writing – review and editing (supporting). **Susumu Takeuchi:** Writing – review and editing (supporting). **Masaru Matsumoto:** Writing – review and editing (supporting). **Kaoru Kubota:** Writing – review and editing (equal). **Kazuo Kasahara:** Writing – review and editing (supporting). **Masahiro Seike:** Conceptualization (equal); methodology (equal); project administration (lead); supervision (lead); writing – review and editing (lead).

## FUNDING INFORMATION

This work was not supported by any specific grant from funding agencies in the public, commercial, or not‐for‐profit sectors.

## CONFLICT OF INTEREST STATEMENT

Takehiro Tozuka has received honoraria from CHUGAI PHARMACEUTICAL and AstraZeneca. Rintaro Noro has received honoraria from CHUGAI PHARMACEUTICAL, AstraZeneca, Merck Pharmaceutical, Pfizer Pharmaceutical, Meijiseika Pharmaceutical, GlaxoSmithKline Pharmaceutical, Daiichi Sankyo Pharmaceutical, and has received Fund for the Promotion of Joint International Research (Fostering Joint International Research), and Grant‐in‐Aid for Scientific Research (C). Akihiko Miyanaga has received honoraria from AstraZeneca, Nippon Kayaku, Merck Pharmaceutical, Kyowa Kirin and Pfizer, inc. Kaoru Kubota has received honoraria from Bristol Myers Squibb Japan, Daiichi Sankyo, Boehringer Ingelheim, Taiho Pharmaceutical, Lilly Japan, MSD Oncology, Chugai Pharma, AstraZeneca, Nihonkayaku, Takeda, and Pfizer, and has received research funding from Daiichi Sankyo (Inst), Boehringer Ingelheim (Inst), Taiho. Pharmaceutical (Inst), and Ono Pharmaceutical (Inst). Kazuo Kasahara has received honoraria from MSD, AstraZeneca, Chugai Pharmaceutical, Bristol Myers Squib, Taiho Pharmaceutical, Pfizer, Eli Lilly, and Boehringer Ingelheim, and has received consulting fees from Chugai Pharmaceutical, Taiho Pharmaceutical, Eli Lilly, AstraZeneca, and has patents with Boehringer Ingelheim, and serves on boards for AstraZeneca and Eli Lilly. Masahiro Seike has received honoraria from AstraZeneca, Takeda Pharmaceutical, Bristol Myers Squibb, Pfizer, Nihon Kayaku, Kyowa Kirin, Ono Pharmaceutical, and MSD, Chugai Pharmaceutical, Taiho Pharmaceutical, Eli Lilly, and Boehringer Ingelheim, and has received research funding from Chugai Pharmaceutical, Taiho Pharmaceutical, Eli Lilly, and Boehringer Ingelheim. All remaining authors report no conflict of interest.

## ETHICS STATEMENT

The study protocol was approved by the Ethics Committee of Nippon Medical School (approval number B‐2022‐544). Informed consent of using the clinical data for this study was obtained by the method of opt‐out on the website from the patients according to instruction by the Ethics Committee of Nippon Medical School.

## Supporting information


Figure S1

## Data Availability

The datasets analyzed in the present study are not publicly available but are available from the corresponding author on reasonable request.
