# Supplementary figures and images for: Osimertinib early dose reduction as a risk to brain metastasis control in EGFR‐mutant non‐small cell lung cancer
Source: Cancer Med. 2023 Sep 11;12(17):17731–9. doi: 10.1002/cam4.6393 (PMC10524078; doi:10.1002/cam4.6393)

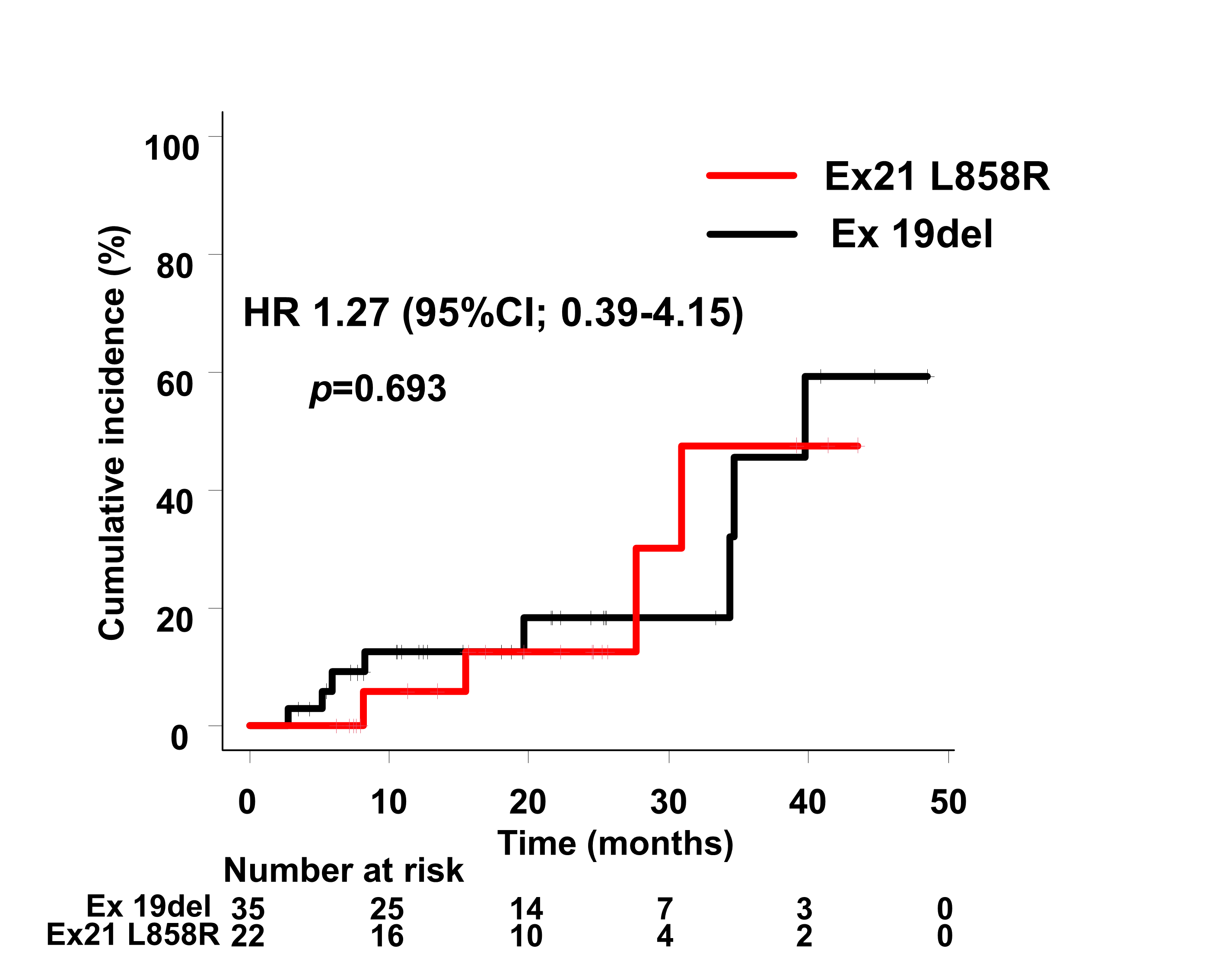

Supplement: Supplementary file 1 — Figure S1 [file CAM4-12-17731-s001.tif]
